# Conserved Binding Regions Provide the Clue for Peptide-Based Vaccine Development: A Chemical Perspective

**DOI:** 10.3390/molecules22122199

**Published:** 2017-12-12

**Authors:** Hernando Curtidor, César Reyes, Adriana Bermúdez, Magnolia Vanegas, Yahson Varela, Manuel E. Patarroyo

**Affiliations:** 1 Colombian Institute of Immunology Foundation (FIDIC Nonprofit-Making Organisation), Bogotá 111321, Colombia; hernando.curtidor@urosario.edu.co (H.C.); cmreyessa@unal.edu.co (C.R.); adriana.bermudez@urosario.edu.co (A.B.); magnolia.vanegas@urosario.edu.co (M.V.); yfvarelaq@hotmail.com (Y.V.); 2 School of Medicine and Health Sciences, University of Rosario, Bogotá 111321, Colombia; 3 Faculty of Health Sciences, Applied and Environmental Sciences University (UDCA), Bogotá 111321, Colombia; 4 Faculty of Medicine, National University of Colombia, Bogotá 111321, Colombia

**Keywords:** synthetic peptides, therapeutics, immunogenicity, malaria vaccine, structure

## Abstract

Synthetic peptides have become invaluable biomedical research and medicinal chemistry tools for studying functional roles, i.e., binding or proteolytic activity, naturally-occurring regions’ immunogenicity in proteins and developing therapeutic agents and vaccines. Synthetic peptides can mimic protein sites; their structure and function can be easily modulated by specific amino acid replacement. They have major advantages, i.e., they are cheap, easily-produced and chemically stable, lack infectious and secondary adverse reactions and can induce immune responses via T- and B-cell epitopes. Our group has previously shown that using synthetic peptides and adopting a functional approach has led to identifying *Plasmodium falciparum* **conserved** regions binding to host cells. Conserved high activity binding peptides’ (cHABPs) physicochemical, structural and immunological characteristics have been taken into account for properly modifying and converting them into highly immunogenic, protection-inducing peptides (mHABPs) in the experimental *Aotus* monkey model. This article describes stereo–electron and topochemical characteristics regarding major histocompatibility complex (MHC)-mHABP-T-cell receptor (TCR) complex formation. Some mHABPs in this complex inducing long-lasting, protective immunity have been named immune protection-inducing protein structures (IMPIPS), forming the subunit components in chemically synthesized vaccines. This manuscript summarizes this particular field and adds our recent findings concerning intramolecular interactions (H-bonds or π-interactions) enabling proper IMPIPS structure as well as the peripheral flanking residues (PFR) to stabilize the MHCII-IMPIPS-TCR interaction, aimed at inducing long-lasting, protective immunological memory.

## 1. Introduction

It has been suggested that acquiring natural immunity against severe clinical malaria in endemic regions [1], protection via immune IgG passive transfer [2,3], immunization (in mice, primates and humans) with live-attenuated sporozoites (Spz) (via mosquito bite, intravenous injection, etc.) [4,5,6,7,8,9,10] and immunization with defined antigens (recombinant proteins or peptides) [11,12,13,14,15] can induce a protective immune response against challenge with live Spz, and/or natural or experimental infection, thereby supporting the feasibility of developing effective malarial vaccines. There is no in vitro culture system for live-attenuated Spz at present, thereby hindering this methodology’s large-scale application using irradiated malaria-infected mosquitoes; multiple mosquito bites do not constitute a proper immunization routine. Some antigens or biological approaches induce short-lived immune responses and the resulting protective immunity is usually species-specific. A completely protective vaccine is still not available, even though different strategies have been followed to date and supposedly promising vaccine candidates have been tested (alone or mixed), making malaria one of the greatest public health challenges [16]. Designing and developing a subunit vaccine (i.e., peptide-based on Spz- and merozoite (Mz)-derived antigens) has thus been suggested as a likely alternative approach.

## 2. A Synthetic Peptide-Based Vaccine Is Feasible

**Figure 1:**
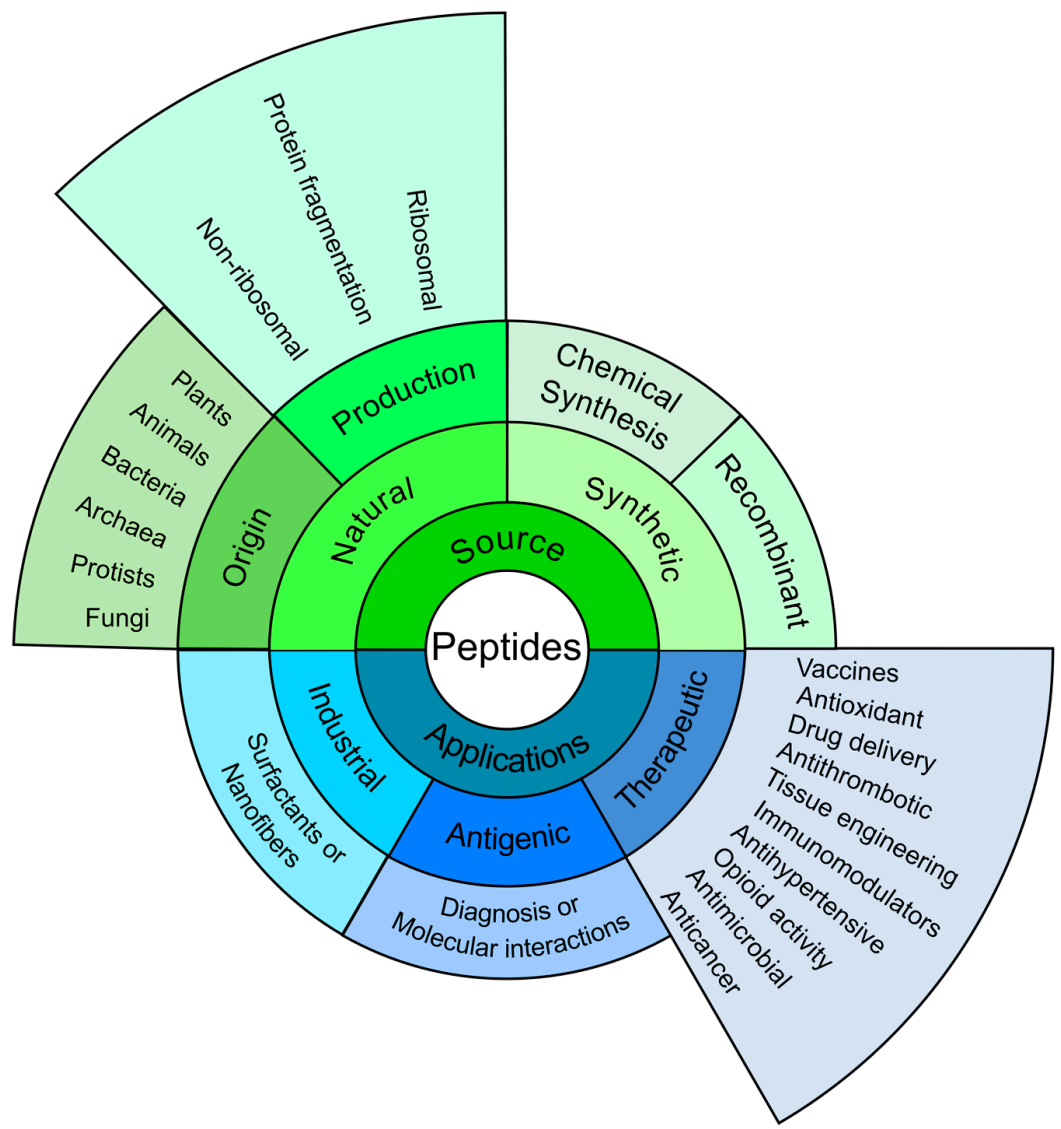
Peptides overview: The figure summarises the main aspects related to peptides: origin, classes and applications. As can be observed, although having some industrial applications [19,20], synthetic peptides are also used as antigens in diagnosing diseases [21,22], studying molecular interactions [23], and identifying pathogens proteins’ functional regions [24,25,26]. However; it is worth highlighting their scope in the vast field of therapeutic applications [27,28,29,30,31,32,33,34].

It was reported more than 50 years ago that a tobacco mosaic virus protein-derived peptide fragment had been able to induce an antiviral response [17]; Merrifield reported the first synthesis of a tetrapeptide in the same year (1963) and coined the concept of stepwise solid-phase peptide synthesis [18]. This led to many papers being published, describing synthetic peptide use for many applications (Figure 1).

Remarkably, synthetic peptides can be used for studying the immune response and its nature, as well as identifying T- and B-cell epitopes or vaccine candidates and their relationship with an observed immune response [11,35,36,37,38,39,40]. It has been shown that using synthetic peptides provides advantages over recombinant fragments; for example, synthetic peptides do not integrate into an immunized host’s chromosomes, immune responses are focused on specific epitopes from one or different antigens into one antigenic formulation and an induced protective immune response is sometimes higher than that induced by other antigens, like live-attenuated or killed parasite vaccines [11,41].

Several reports in the 1980s highlighted the success achieved of using synthetic peptides as vaccines against several diseases caused by different types of pathogen and in different models (Table 1). Our group found that immunising *Aotus* monkeys with a synthetic chimera (SPf66), using circumsporozoite surface protein (PfCSP)-derived immunodominant NANP repeat sequence as spacer [42,43], induced fully-protective immunity in 50% of them and a similar percentage in human volunteers against experimental challenge with infected erythrocytes [11,39,44]. This was the first antimalarial, multi-epitope and multi-stage synthetic vaccine ever developed [11].

Several Phase IIb and Phase III clinical trials, showed that SPf66 was safe and highly-immunogenic in humans and protective (30–40%) in semi-immune populations subject to natural challenge and in several independent randomized controlled trials (Table 1). However, SPf66 efficacy decreased in a trial involving the newborn and infants when delivered through Tanzania’s expanded programme of immunization (EPI) [64,65,66]. We thus decided to stop SPf66 vaccination and focused our efforts during the next 20 years on developing a complete, fully protective, second-generation malaria vaccine.

## 3. Adopting a Functional Approach for Vaccine Development

**Figure 2:**
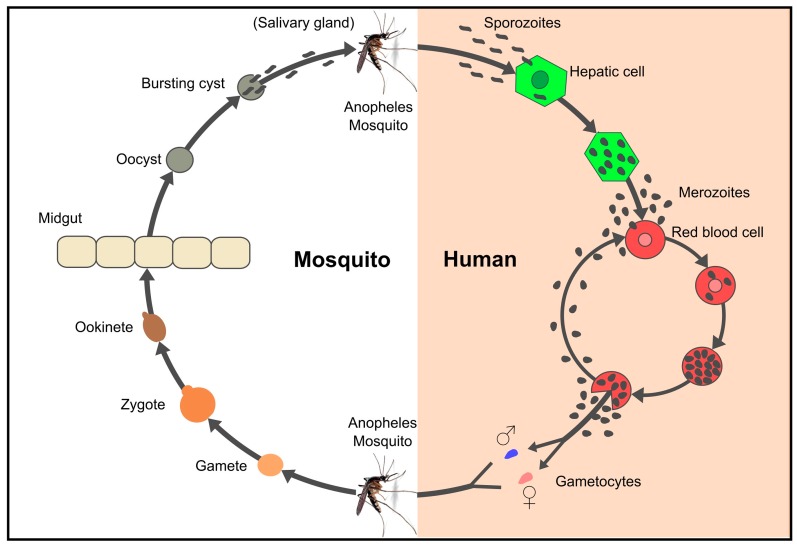
Life cycle of *Plasmodium falciparum.* Spz entry through the bite of a female *Anopheles* mosquito begins the *Plasmodium* spp. life-cycle in humans. Spz travel through the bloodstream to reach and invade liver cells, thereby initiating the pre-erythrocyte cycle [70]. Spz grow and divide within the hepatocytes, producing 10,000–30,000 Mz per invaded hepatocyte, depending on the *Plasmodium* specie [71]. Once Mz are released into the bloodstream, each one can invade RBCs in about 45 s. Mz develop to ring, trophozoite and (48 to 72 h) schizont stages inside RBC, producing about 8–32 Mz which can invade new RBC [72]. Some Mz develop into sexual forms called gametes (male and female) which are ingested by a mosquito. Sexual reproduction takes place within a mosquito’s midgut where new Spz migrate to the salivary glands to start the parasite’s life-cycle all over again [73].

*Plasmodium* parasites have a complex life-cycle involving two hosts (humans and *Anopheles* mosquitoes), three invasive stages (Mz, Spz and ookinete) and three different host cells which are the target for such invasive stages: Mz invade red blood cells (RBC), Spz hepatocytes and ookinetes epithelium midgut cells (Figure 2). Around 80 proteins have been shown to be involved in the invasion of these susceptible cells [67,68,69].

The parasite’s life-cycle complexity is just one challenge to be understood in depth, as it has developed different evasion mechanisms, such as protein variation, antigenic polymorphism, altered peptide ligands (APLs), alternative invasion pathways, cryptic epitopes, immunosuppression, immunological smoke-screens and antigen shedding, thereby inducing long- or short-lived non-protective immune responses [74,75,76,77,78,79,80]. Repetitive antigens (used erroneously as vaccines) have been targets for strong, but not protective, immune responses. It is believed that repetitive antigens, such as the circumsporozoite (CS) protein amino acid (aa) sequence, acts as an immunological “smoke-screen” helping to evade the immune response [81,82]. All the above has suggested that using antigens (or regions derived therefrom) as targets for natural antigenicity should be avoided when selecting vaccine components.

Successful SPf66 results suggested that peptide length was suitable and that peptide polymerization, increasing peptide (antigen) length, was a good option regarding synthetic vaccine development [11,12,64,83]. However, it was also evident that more antigens (subunit components), representing proteins from the main stages in the parasite’s life-cycle (Mz and Spz), had to be included to ensure a fully protective, anti-malarial vaccine; however, the question remained how many should be selected and how should this be ascertained?

## 4. Subunit Component Selection

Different approaches have been adopted for selecting the best candidates for use as vaccines. The first antigens (pre-genome era) were whole inactivated/killed parasites [84,85,86,87,88] or were selected following the so-called **immunological approach**, i.e., proteins or their regions recognized by immune sera from adults or infected monkeys. Such epitopes can induce a protective immune response, or are the target for monoclonal antibodies which, in turn, can inhibit asexual blood stage growth in vitro [89,90,91]. Other antigens were selected for their structural characteristics [92], their location on Mz or Spz surface, or for playing a critical role in parasite development or Mz invasion of erythrocytes [93,94,95]. More recently, data obtained following publication of the *Plasmodium falciparum* genome (post-genome era), transcriptome and proteome has led to around 80 proteins being identified as key elements in RBC and hepatocyte invasion [67,68,96,97,98,99,100].

While studying SPf66-induced humoral immune response in humans [101,102] and looking for new vaccine candidates [103,104] we were also studying the relationship between Mz proteins and RBC invasion. It was seen that peptides having high RBC binding activity could also inhibit Mz invasion of RBC; remarkably, two out of three Mz protein-derived peptides (included in the SPf66 vaccine) were found [105]. This pivotal observation led to the breakthrough from using the classical immunological approach to searching for vaccine candidates, involving a completely **functional approach** which we have followed during the last 30 years. Mz- and Spz-derived high activity binding peptides (HABPs) associated with the invasion of both host cell types had to be recognized.

A highly robust, specific, sensitive and well-designed receptor-ligand binding methodology [106,107,108,109], was used to recognise functionally-relevant aa sequences in all proteins described as being involved in RBC or hepatocyte binding and invasion. Entire protein sequences were synthesized as sequential 20-mer-long peptides and their ability to bind hepatic cells or RBC was determined. Hill coefficients (*n_H_*), dissociation constants (*K_d_*) and the amount of binding sites were calculated for those having high binding activity (i.e., HABPs), as well as the nature and molecular weight of their receptors [110,111,112]. Some Mz-derived HABPs inhibited Mz invasion of RBC, suggesting an association with critical parasite functions, such as recognition, rolling, reorientation, gliding and/or attachment and invasion [107,111,113]. Using these peptides as subunit components of an antimalarial vaccine thus gained wide recognition (i.e., highlighting the value of adopting a functional approach).

Such rationale was substantiated by immunising *Aotus* monkeys with polymeric HABPs and experimentally challenging them with freshly-obtained RBC infected by the highly-infectious *Aotus*-adapted *P. falciparum* FVO strain. The apparently disappointing results showed that HABPs having conserved aa sequences (cHABPs) were poorly or non-immunogenic and did not induce any protection in the *Aotus* monkey model. However, HABPs having variable sequences (vHABPs) could induce a highly strain-specific protective immune response [114,115,116,117]. These results agreed with reports showing that antigenic or immunogenic proteins having variable aa sequences had been used as an immunological evasion mechanism (i.e., they induced strain-specific immune protection and repeat aa sequences induced non-protective immune responses) [76,81,118]. These results partly explained the systematic failure arising from using complete X-ray-irradiated or genetically-altered parasites, DNA naked encoding specific proteins or complete recombinant proteins, since practically all of them have been seen to have genetic variability in one or several regions [119,120,121].

BLAST analysis of the aforementioned results regarding all *P. falciparum* strains described to date suggested that cHABP regions lacking genetic variation or polymorphism in their aa sequences should involve critical biological functions for parasite survival, like maintaining protein folding, acting as ligands during invasion [113,122], have metal (Mg^++^, Ca^++^) or ATPase binding, enzymatic activity (phospholipase or serine protease), cavity formation, Ca^++^ release, membrane transport and protein export via *Plasmodium* export element (PEXEL) motifs [113]. This might explain why such sequences are immuno-silent and, therefore, the perfect vaccine target, or in our words, “the malaria parasite’s Achilles heel” [123].

Our results showed that the parasite avoids the immune response by using conserved aa sequences to perform critical biological functions. Although these sequences are available for the immune system, they are immunologically silent due to the particular and specific 3D structure they adopt [123,124]. Furthermore, protein 3D studies have shown that parasite cHABPs from the most functionally-relevant structures involved in host cell invasion are craftily located far away from highly polymorphic and immunogenic regions to distract the immune response [79].

Based on the altered peptide ligand concept (APL), where some aa replacements modulate the immune response, we decided that conserved functional sequences had to be modified to break the parasite’s immunological code of silence, following a clearly established set of rules [77,125]. Genetic control of the immune response had to be taken into consideration as we had been warned long beforehand by analysis of the immune response induced in individuals immunized with SPf66. This data showed an association of low or non-responders with a particular MHC class II allele (HLA-DRβ1*0401), suggesting imperfect SPf66 vaccine component fit into the MHCII-peptide-T-cell receptor (TCR) trimer complex [101,102], as well as preferential usage of TCR Vβ3 or VβII regions having a similar aa sequence to that of cHABP 1513 included in SPf66.

Non-responsiveness and/or non-protection-inducing ability led to two very relevant characteristics: imperfect fit into MHCII molecules (since it was later shown that cHABP 1513 had high HLA-DR1*0801 binding capability) and inducing a TCR preferential usage having high aa sequence homology with the 1513 cHABP [126].

Studies based on the elegant description of MHC genetics, function, 3D structure and role in regulating TCR antigen recognition on T-cell membrane [127,128,129] have opened the way forward for the rational development of vaccines. This has involved identifying antigens recognized by specific MHC molecules able to form stable MHC-peptide-TCR trimer complexes and thus induce a suitable immune response; however, as the human MHC is highly polymorphic, specific antigens and the amount of determinants necessary to overcome MHC restriction [130] have had to be carefully selected to avoid a non-responsive, suppressive or harmful immune response.

Since *Aotus* monkeys are highly susceptible to human malaria, their immune system molecules are very similar (80–100% identical) to that of humans and their experimental infection can be treated with antimalarial drugs [131,132,133,134,135,136] they thus form an ideal experimental model for malaria research. This meant that conserved binding regions in the most relevant proteins involved in parasite-host cell invasion and infection had thus to be identified from a biological point of view and their 3D structure determined from a structural and physicochemical point of view. Converting such key regions (cHABPs) into immune protection-inducing protein structures (IMPIPS) [63,77,123] thus became the driving force behind our logical and rational methodology for vaccine development, using *P. falciparum* malaria as our prototype disease.

## 5. Breaking the Parasite’s Immunological Code of Silence

Different strategies have been proposed for enhancing short peptides’ immunogenicity (i.e., natural or synthetic adjuvants or delivery system), such as using carriers [137,138,139,140], conjugation with T-helper and B-cell activation proteins [139], identifying cryptic epitopes [141], peptides mimicking native structures (Cys–Cys bond inclusion) [142], peptide elongation [143] and using MHCII peptide binding prediction servers for predicting new epitopes [144]. Most of the above approaches involve modifying peptide or epitope sequences so that MHC molecule and/or peptide-MHC complex binding affinity for TCR molecules becomes increased [145].

The seminal finding which led to us modifying cHABPs arose from the observation that, when the long (40-mer) linear, polymerized SPf66 molecule was synthesized (~10 KDa), the methionine residue in the 83.1 peptide sequence (YSLFQKEKM*VL) became converted into sulphomethionine due to long-lasting, recurrent deprotection treatment; this did not occur with the 83.1 short monomer (12-mer), having high RBC binding capability [107]. This particular residue modification changed peptide polarity and its 3D structure. It thus became clear that cHABP physicochemical and structural characteristics would have to be modified to render them highly immunogenic and protection-inducing. HABP analogues were synthesized and tested in binding assays to identify “critical” binding RBC residues in an attempt to break the parasite’s immunological code of silence associated with functionally relevant cHABP sequences, since methionine was a critical binding residue in cHABP 83.1 (glycine scanning) [111,115,146,147]. As in a previous report determining the particular contribution to antigen-antibody interaction of each residue in an antigen sequence [148], we found that some residues could be “critical” for cell binding [111,115,146,147].

When modifying critical binding residues in cHABP sequences, for increasing their immune response [149], we found that some mHABPs did not induce any immune response or protection after experimental challenge while others induced high antibody titers (short-lived or long-lived) but did not induce any protective immune response [115,116,150]. Interestingly, some mHABP sequences induced high, long-lived antibody titers, conferring complete, sterile protection against experimental challenge in *Aotus* monkeys [115,116,151]. The results for cHABP derived from the same or different proteins [114,146,152,153], after analyzing many synthesized peptides (~40,000) and a suitable/statistically significant amount of monkey immunization assays revealed that critical binding residues had to be replaced by others having similar mass, volume and surface, but opposite polarity (i.e., F by R and vice versa R; W↔Y; L↔H; P↔D,N; M,L↔K; A↔S; C↔T or V↔Q,E), one of the fundamental rules in synthetic vaccine development [111,151,154,155].

^1^H-NMR (500 or 600 MHz) determination of three-dimensional (3D) structures and cHABP and mHABP binding to purified HLA-DRβ1 human molecules highlighted the fact that careful cHABP sequence changes induced structural modifications thereby allowing better mHABPs-MHC interaction and induced protection for *Aotus* monkey against experimental challenge with the highly infectious *P. falciparum* FVO strain [155,156].

Once questions related to antigen selection and how to increase immunogenicity and protection-inducing capacity had been resolved; the next challenge lay in identifying the mechanism by which mHABPs could induce a protective immune response. As expected, increased mHABP immunogenic properties were closely related to their 3D structure and their interaction with MHCII and TRC molecules. All this was related to mHABP sequences’ intimate physicochemical features: length, charge, volume, secondary structure, aa side chain orientation, intra-molecular interactions and peripheral flanking residue (PFR) secondary structure.

## 6. Chemical Features to Be Taken into Account in Vaccine Development

### 6.1. Immune System Molecules’ 3D Structure

MHC molecules were so named by Gorer and Snell’s pioneering work due to their prominent role in histocompatibility acceptance or tissue and/or organ rejection during transplants [157,158]. Located on antigen-presenting cell surface, they are also responsible for a host adaptive immune response by presenting short derived peptides to the TCR [159]. An in-depth understanding of the MHC-antigen-TCR triad is thus needed for inducing antibodies against foreign intruders and disease control.

**Figure 3:**
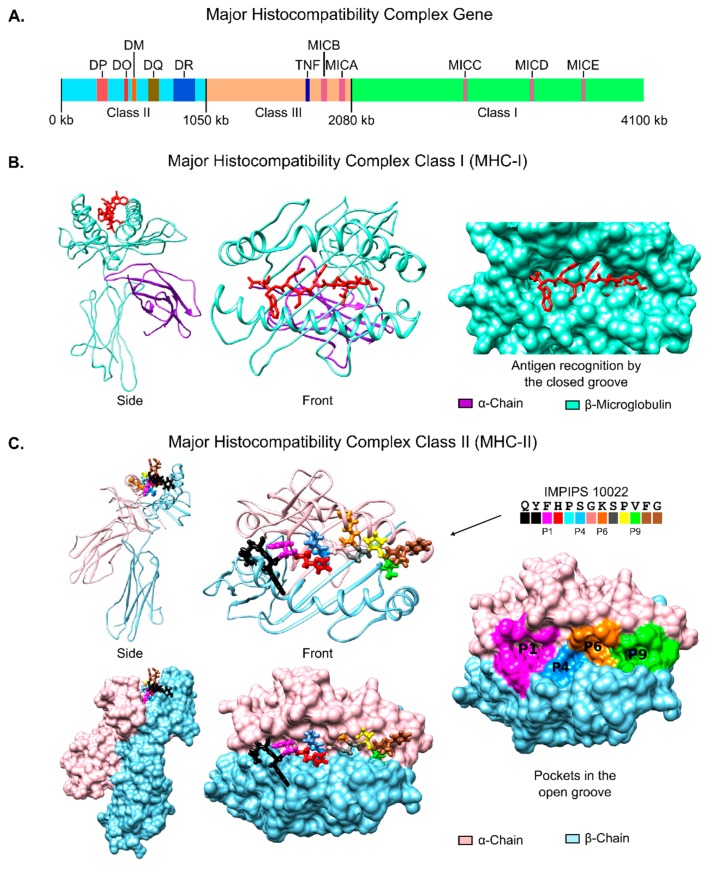
Major histocompatibility complex. (**A**) Schematic representation of the gene encoding the three MHC classes, with the most important proteins roughly located; (**B**) Major histocompatibility complex class (MHC) I ribbon and surface structure (based on PDB code 5MEP) displaying the α-chain, the β-microglobulin as well as the closed groove for peptide antigen recognition [166]; (**C**) MHC class II ribbon and surface structure (based on PDB code 1A6A [167] displaying the α- and β-chains and the open groove for peptide antigen (IMPIPS 10022, sequence and colour code given) recognition [168] coloured according to the same color code for pockets P1, P4, P6 and P9.

The MHC gene encodes three different molecules (class I, II and III (Figure 3A); MHC class I and II play relevant roles in antigen presentation while MHC class III encodes complement system molecules and inflammatory cytokines, including tumor necrosis factor (TNF) [160,161,162].

MHC class I consists of two non-covalently linked α- and β2-microglobulin (β2m) chains, the α-chain being highly polymorphic and the β2m-chain conserved. This MHC molecule binds 8–10 aa-long peptides (either self- or non-self) in endoplasmic reticulum lumen. The MHCI-peptide complex (Figure 3B) travels to the cell surface to present intracellular antigens to the CD8^+^ cytotoxic cell TCR after binding, involving several molecules, such as transporter associated with antigen processing (TAP), tapasin, calreticulin, calnexin and the thiol oxidoreductase of the endoplasmic reticulum (Erp57) [163,164,165].

MHC class II molecules are normally found only on professional antigen-presenting cells, like macrophages, B-cells and dendritic cells (DCs). They bind processed antigens (resulting from protein acidic–enzymatic cleavage) in the endosomal-lysosomal compartment with the help of a non-classical class II molecule (HLA-DM) to become externalized on cell surface for recognition by TCRs on CD4^+^ T-lymphocytes [165,169]. T-cell memory and effector cell differentiation are mediated by MHC class II; thus memory cells become activated when the same antigen/infection enters an organism and the immune response is triggered [169]. Therefore, a complete understanding of antigen presentation is required for vaccine-development; structural MHC class II features have thus to be established since it is known that an antibody-mediated malaria immune response is highly associated with class II molecules [170,171,172,173]. Very elegant structural analysis has shown that multifunctional MHCII molecules are formed by two non-covalently associated and cell membrane-anchored proteins [127,128,129,130,131]. The peptide groove or binding region (PBR) in distal α1 (almost conserved) and β1 domains (polymorphic) is formed by a pair of opposite α-helices on a floor of eight antiparallel β-strands (Figure 3C).

The PBR is open at both ends and can bind 13–25 residue-long peptides; however, the interaction involves only 9 residues by means of four major pockets (numbered relative to the largest HLA-DR1 pocket 1 (P1), accommodating and stabilizing the peptide side chains of the residues located in positions (p) p1, p4, p6 and p9 (Figure 3C). Pocket polymorphism explains class II allele specificities; several combinations of the aforementioned four aa are permitted, since all four pockets can accommodate a wide variety of side chains. An antigen’s overall specificity depends on the four residues’ proper fit into such pockets; the peptide backbone should have a zig-zag like structure, similar to a polyproline type II conformation and, since the immune response relies on a suitable MHCII-antigen-TCR interaction, antigen recognition is restricted by the characteristic sequence of each chain forming the heterodimeric MHC II molecule [174,175].

We found that Mz apical membrane antigen 1 (AMA-1) cHABP 4313 (^134^DAEVAGTQYRLPSGK**C**PVFG^153^)-derived mHABP 10022.43 (DAEVAGTQYF^p1^HPS^p4^GK^p6^SPV^p9^FG) having two left-handed PPII_L_ regions in p1–p4 and p6–p9, experimentally bound to HLA-DRβ1*0302, HLA-DRβ1*0701 and HLA-DRβ1*0301 purified molecules. Such structural conformation allowed mHABP 10022.43 an almost perfect fit into HLA-DRβ1*0302 (Figure 3C), [176,177]. These alleles had a dimorphic sequence in P1 allowing different residues to fit into it. Such observations were also made for P4, P6 and P9, meaning that MHCII–peptide interaction (and thus the immune response) was dependent on the stereo-electron characteristics of the residues forming genetically-determined pockets [62,178]. MHC class II architecture thus implies that peptides must be at least fifteen amino acids long to induce the appropriate immune response regarding any peptide-based vaccine.

### 6.2. Charge and Volume

**Figure 4:**
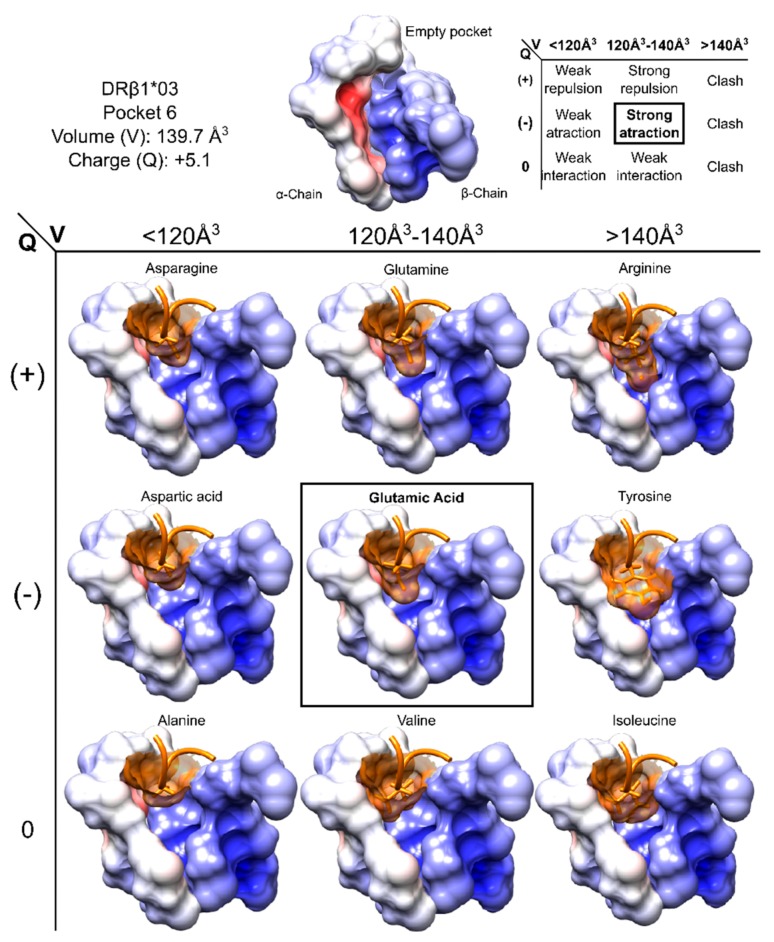
Pocket volume and charge showing aa having different volumes and charge fitting into HLA-DRβ1*03 P6. Pocket characteristics and interactions are summarised at the top of the Figure, while the surface structures of aa occupying P6 are shown at the bottom. P6 surface is coloured according to its electrostatic potential set from −3 *kT/e* (negatively charged, red) to 3 *kT/e* (positively charged, blue). Figure based and adapted from [187]. Reproduced with permission from Patarroyo ME, *Biochem. Biophys. Res. Commun.*; published by Elsevier, 2017.

Stable interactions in the MHC-peptide-TCR ternary complex are needed to trigger an appropriate immune response and (like any other physical phenomenon) it has its own features and requirements, charge and volume being crucial for such interaction. Every antigen aa interaction with MHC and TCR molecules has to maximize attraction forces, as well as minimize repulsion forces. There are ideal values concerning the maximum volume accepted (related to steric hindrance) and electron characteristics (according to attracting forces’ formation) regarding every position. Figure 4 shows HLA-DRβ1*03’ P6 where smaller aa elicit weak interaction forces whilst bigger ones do not fit into this relatively small pocket. The interaction forces for aa having the proper volume will depend on the side chain charge. Each human MHCII molecule has conserved and specific electrostatic characteristics, correlated with the peptide sequence with which they interact [179]. Protein (MHC and TCR)-peptide interactions can thus be seen as the total sum of interactions between aa and such interactions are ruled by the specific characteristics of each of the 20-mer aa and their possible combinations.

A theoretical analysis of aa interactions (200 possible aa pairs) based on three simple premises (opposite charges attract while similar charges repel, convex fits to concave and similar hydrophobicity works together), based on size, charge and hydropathy indices for protein structure analysis, revealed the prevalence of opposite-charged residue pairs, along with unexpected ones, like positive-neutral pairs being more compatible than negative-neutral pairs [180]. Another study, using discrete molecular dynamics (DMD), evaluated peptide-protein recognition in a set of ten protein–peptide systems. A two-step binding mechanism model was proposed which included random peptide-protein region collisions to form a metastable “encounter complex”, the second step being peptide docking on protein surface which required conformational receptor change to reach a native-like binding state. These results suggested that electrostatic interactions played an important role in peptide-protein recognition and highlighted the importance of appropriately charged residues in peptides used in vaccine design [181,182].

A study involving quantum chemical topological (QCT) calculation of Becke-3 parameter-Lee-Yang-Parr charges (polarisation-consistent (pc) double-ζ plus polarization basis set (B3LYP/apc-1)) located protonated (R, K and H) and deprotonated (D and E) residue charges [183,184]. Comparing average atomic charges for these five residues (over a thousand conformations for both charged and neutral aa) revealed that 81% of negative charges were located on the aspartic acid side chain while 88% were on the glutamic side chain, whilst the entire charge for the protonated ones was located on the side chain. This led to the conclusion that the peptide backbone was not greatly influenced by side chain charge due to methylene group insulator effect in these five aa, as well as when charge was spread over several atoms (e.g., H and R) [183].

Studies regarding the role of specific peptide residues binding to MHCII-PBR during antigen presentation have shown this complex system’s sensitivity to small changes in aa charge, volume and orientation or regarding peptide structure [185]. An aa peptide-MHC binding prediction-derived similarity matrix has shown that the most unfavorable effect of a single residue substitution results from substituting residues having opposite charges [186].

For example, our MHC-DR studies have shown that DR8 P6 (deep and negatively charged) allowed positively-charged residue binding in the MSP-1^38–55^ peptide (a SPf66 sequence extension) and stimulated human T-cell response [11,80]. We found that P4 in DR4 and DR3 was also important for determining DR-bound peptide side chain specificity, binding negatively-charged side chains and having an alternative binding register [80].

Recent in-depth structural analysis has shown that any malaria vaccine component identified as highly immunogenic and protection-inducing (following challenge in immunized *Aotus* monkeys) can be readily used in humans without further modification [187]. Since *Aotus* MHCII 3D structure has not yet been described, we used well-known HLA-DRβ1* crystallographic structures to model monkey alleles. By comparing electrostatic potentials and volume calculations we were able to establish differences between human and monkey MHCII molecules, concluding that such differences were not great enough to prevent any vaccine candidate component developed from the *Aotus* model being readily used on humans [187].

The forgoing means that aa in positions p1, p4, p6 and p9 (for any peptide-based vaccine) must be carefully chosen to maximise the interaction with MHC-II molecules and induce an appropriate immune response.

### 6.3. Peptide–Antigen Length

Even though both MHCI and MCHII molecules bind antigens to present them to the TCR and trigger an immune response, there are huge structural differences between these two molecules’ PBR which must be taken into account during vaccine development. MHCI molecules comprise a closed binding site, allowing them to accommodate 8–10 residue-long peptides, while MHCII molecules can present much longer peptides due to their open-ended PBR [175,188]. Peptide elongation (greater than 9-mer long) can increase HLA-antigen affinity (optimal 18–20 aa peptide length); however, the effect becomes negative beyond this point, being associated with enzyme degradation [143,189].

We found that the distance of the nine aa fitting into the PBR groove was actually more important than the amount of aa in a peptide. Our studies of MHCII molecules, ^1^H-NMR antigen structure and antigen-interacting active site characteristics concluded that any antigen must have a 26.5 ± 1.5 Å distance between the first and ninth aa fitting into the PBR [62].

### 6.4. Polyproline as Preferred Secondary Structure

Antigen-interacting MHCII groove characteristics imply that a proper interaction between MHCII and an immunogenic peptide arises from appropriate antigen conformation. Two decades ago it was found that secondary structure of an antigenic (haemagglutinin A, HA)-derived peptide bound to HLA-DR1 had similar structure to that of a polyproline type II (PPII_L_) left-handed helix [190,191]; since then, many studies with experimental immunogens in mice, and others with the human antigen-HLA-DR complex, have reached the same conclusion.

The PPII_L_ helix, having three residues per turn (3.12 Å per residue), results in a considerably extended helical structure which commonly occurs in globular proteins [192,193,194]. It has a trans isomer for the peptide bonds, a left-handed helix, no hydrogen bonds and φ = −75 ± 25° and ψ = 145 ± 25° dihedral angles. P, Q and D are common residues in PPII_L_ conformations, accompanied by some aliphatic residues (G, A and L), also having high propensities for left-handed polyproline II helix formation [195]. Typical PPII_L_ CD spectra in water are characterized by a negative band around 200–205 nm and a weak positive band at 217–225 nm [196,197]. Due to its importance in protein–protein and peptide–protein recognition, it has been proposed that PPII_L_ should be regarded as being equally important as classical α-helix, β-sheet and β-turn secondary structures [198,199].

Our group has shown that mHABPs having total or partial polyproline II-like left-handed (PPIIL) helixes establishing H-bond (~7–11) interactions with specific residues in MHCII are highly immunogenic protection-inducers or are very high, long-lasting, antibody-inducers [62,200]. Peptides must thus have PPIIL-like secondary structure to fit perfectly into MHC-II molecules and trigger a protective immune response.

### 6.5. Amino Acid Side chain Orientation

Thinking about protein or peptide secondary and tertiary structures usually concerns phi (φ) and psi (ψ) backbone angles; however, for a given aa having fixed φ and ψ angles, there is another very important (yet underestimated) angle known as chi1 (χ^1^) (except for glycine, alanine and proline), defined as the dihedral angle involving rotation around the Cα-Cβ bond. This is the angle greatly determining a peptide or protein’s entire side chain orientation [201,202]. The χ^1^ variation of a given aa regarding a peptide-antigen implies a change in its side chain orientation, thereby directly affecting interaction with either the MHCII or TCR molecules, leading to a change in the complex’s affinity and/or stability [178].

After an in-depth examination of 61 globular proteins in the search for a relationship between side chain orientation and secondary structure, McGregor et al. found that χ^1^ had a trimodal distribution in globular proteins adopting a trans (to the amide group, χ^1^ around 180°), *gauche*^+^ (trans to the carboxyl group, χ^1^ around 300°) or *gauche*^−^ (trans to the α-hydrogen, χ^1^ around 60°) orientation [203]. The preference was found to be for the *gauche*^+^ rotamer due to the steric clash between Cγ and the backbone in the other two rotamers, and almost-full exclusion of the *gauche*^−^ rotamer for the α-helix due to (i, i-3) steric clashes [203]. Later on, 70 polypeptides’ aa rotamericity (defined as the percentage of residues in the ±20° expected rotamer values interval) was evaluated by Schrauber et al. [204] when studying side chain conformation and deviation from expected orientation. The study gave 5–30% frequency for this deviation (±20°) depending on the aa, and that such rotamericity depended on secondary structure, backbone torsions and tertiary packing requirements (from non-accessible or internal regions). Their results contradicted the idea that protein interiors are relaxed, proving that globular proteins’ aa side chains are actually strained in some regions, due to preferred whole protein stability [204].

It was known that protein folding involved a loss of entropy, explained by a folded protein’s decreased degrees of freedom compared to those for an unfolded one [205]. Very elegant research by Doig et al. [206] evaluated conformational entropy changes in aa side chains due to protein folding. They calculated the mean of such entropy changes per side chain, using several sets of results, and found that Boltzmann’s equation gave a *T*Δ*S_conf_* −0.95 kcal/mol per residue mean value at 300 K and a *T*Δ*S_conf_* −0.46 kcal/mol per bond mean value or rotable χ angle. Taking differences between states into account, they established a −4.37 kcal/mol per residue mean value as the absolute entropy-change (*TS*). This value contrasted with a −0.6 kcal/mol per rotor mean value at 300 K when considering fusion entropies for some organic compounds which are used as the model for entropy changes in protein folding [207,208]. This study showed the considerable importance of changes in side chain conformational entropy, consensus value being ~−1 kcal/mol per residue regarding protein folding or ~−0.5 kcal/mol per rotamer, implying a huge loss of conformational entropy due just to folding [206].

Our group recently reviewed findings regarding the χ1 angle’s effect on the immune behaviour of 20-mer mHABP vaccine candidates [178]. ^1^H-NMR 3D structures of a set/mixture of peptides having different immunological activities were established and compared to X-ray crystallographic 3D coordinates of different antigens in the PDB-database. This study concluded that a *gauche*^+^ orientation for a residue in p3 fitting into PBR P3 was critical when mHABPs were used in immunization mixtures. It was suggested that this structural feature avoided competition suppression or blocking caused by the mHABP mixture’s presentation, due to proper orientation with the corresponding TCR complementary determining region (CDR). It was also found that the residue in p7 fitting into PBR P7 had to have *gauche*^+^ orientation for inducing fully-protective immunity and long-lasting antibody memory [178]. Thus correct orientation of p3 fitting into PBR P3 and p7 fitting into PBR P7 is essential for residues’ proper interaction with the corresponding TCR CDR region.

### 6.6. Hydrogen Bonds and Intramolecular π-Interactions

Interactions in and between aa are no different from some other attractive or repulsive forces and complete understanding of them is required, since these forces directly influence a given protein or peptide’s structural conformation [209,210]. Hydrogen bonds (H-bonds) are known to be one of the most ancient and have been identified as being one of the strongest non-covalent forces; they are responsible for water’s unique characteristics and are one of the cornerstones of life on earth [211]. As well as being one of the strongest non-covalent forces, H-bonds, along with electrostatic attractions, van der Waals forces and π-interactions, are attractive forces, while steric hindrance and electrostatic repulsions are repulsive ones [212,213,214,215,216].

Backbone H-bond contribution to protein stability was controversial for authors believing that H-bonds were destabilizing forces (since intra-peptide H-bond formation breaks peptide-water H-bonds) [217,218]. Nevertheless, others showed that backbone H-bonds actually stabilized proteins, given short poly-Ala peptides’ observed α-helix propensity at low temperatures [219,220], that buried H-bond disruption destabilized proteins more than a solvent-exposed H-bond could [221] and that environment-dependent H-bonds played a pivotal role in protein folding [182,212].

H-bonds are one of the pillars of protein structure (as recognized today); they are a well-recognized factor in helix (α-, π- and 3_10_), β-sheet and most β-turn formation, as well as other protein conformation components [210,212]. H-bonds in proteins and peptides are usually (but not exclusively) established between the carboxyl group’s oxygen from one residue and the amide hydrogen from another residue [212]. There are several other H-bond-like forms involving polar aa side chains; all of them are directly involved in protein structure stabilization and biological function, recognition and protein-protein interaction [222].

H-bonds’ essential role in protein structure and in immune responses has become evident, since T-cell epitopes’ total retro-inversion (d-aa) has been seen to have very low MHC binding capability and this was non-immunogenic [223]. We have found that specifically replacing l-aa with d-aa in MSP-1-derived HABP 1513 binding motif did not reduce its binding activity, although its secondary structure became distorted, and that d-analogues were less suitable for T-cell stimulation [224,225,226]. By contrast, some d-aa replacements (one at a time) in MSP-1-derived HABP 1585 produce d-analogues having improved resistance against proteolytic degradation and higher binding activity than that the cognate peptide. Interestingly, a d-analogue was able to induce protective immune response in *Aotus* monkey model, suggesting that α-carbon chiral transformation is a critical target for structural modulation of the immune response [227]. This would suggests that d-aa are useful for protecting d-analogue peptides from proteolysis and should be taken into account in peptide-based vaccine design. Further studies are needed for determining the precise steps to be followed for residue replacement and peptide design.

On the other hand, our group’s structural data showed that cHABPs (identical to their recombinant counterparts) mediated receptor-ligand interactions by forming part of H-bond-stabilised channels or troughs and were located far from or opposite to highly polymorphic regions, suggesting an immune escape mechanism [79,228]. It was found that cHABPs became highly immunogenic and sterile immunity inducers in the *Aotus* experimental model when aa establishing H-bonds with own aa, with those from other cHABPs or binding to host cells were modified [77,229,230,231].

Merozoite surface protein 2 (MSP-2)-derived cHABP 4044 (^21^KNESKYSNTFINNAYNMSIR^40^) bound to HLA-DR molecules but did not induce antibodies against *P. falciparum* or protection against experimental challenge in *Aotus* monkeys, while P4-24112 mHABP induced very high antibody titres and complete protection in 66% of immunized *Aotus* carrying the HLA-DR β1*0403 allele [232]. Molecular modelling of mHABP 24112 and docking its structure into HLA-DRβ1*0401 showed the spontaneous formation of 7 H-bonds between its backbone atoms and HLA-DR aa and 3 more with TCR CDR3β [232]. A similar result was found for circumsporozoite protein (CSP)-derived cHABP 4383 (^68^NSRSLGENDDGNNEDNEKLR^87^) [233]; however, it did not bind to any of the HLA-DRβ1* molecules tested here, while mHABP analogue 25608 bound to HLA-DRβ1*0401, inducing a very high immune protection-inducing response [234]. Molecular modelling and docking showed the spontaneous formation of 12 H-bonds when the 25608.37 conformer was superimposed onto HLA-DRβ1*0422 [234].

Cation-π interactions involving electrostatic attraction between aromatic rings (quadrupole created by the π-electron cloud) and a monopole (positively-charged groups) are also one of the most important interactions involved in molecular recognition, catalytic mechanisms and structural biology [216,235,236,237,238]. Sunner et al. made the seminal observation [239] that potassium ions (K^+^) in gas-phase preferentially bound to benzene rather than water and established an axial structure by ion-quadrupole and ion-induced dipole attraction; this stimulated thought concerning the nature of cation-π interactions [216,240]. Impressive work by Burley and Petsko [241,242,243,244] measured, defined and established the importance of protein π-interactions and their impact on tertiary structure. A significant finding stated that an average of 60% of globular proteins’ aa aromatic side chains were present, at least in aromatic pairs (aro-aro interaction), and 80% of them formed clusters of three or more interacting aromatic residues (named π-π interaction). They also described aromatic side chain (F, W and Y) interaction with positively-charged aa (K, R, N, Q and H) (cation-π interaction), as well as oxygen and sulphur atoms interaction with aromatic rings in proteins. Studies with benzene and aa F, W and Y showed this interaction to be an electrostatic effect involving cations and aromatic rings’ quadrupole moment and also that the cation-aromatic interaction could compete with water in solvating cations [240]. This work, together with a study showing that cyclophane aromatic rings recognised a positive charge and that cation–π–cation complex stability (intrinsically repulsive) was largely dependent on the local environment, led to the effect being termed an “ion-dipole” interaction [216].

An average 9.5 kcal/mol stabilisation energy and edge-anion geometry for this interaction was found using benzene, phenol and indole interacting with formate to mimic interactions between aromatic and anionic aa and second order Møller-Plesset perturbation theory (MP2) calculations. A rapid search of selected PDB entries gave an over-representation of nearly-planar geometry, accompanied by under-representation of axial geometry for anionic-aromatic interacting residues, thereby supporting this finding [245]. Later on it was showed that anion-π force geometry seemed to be related to ring electron density since the anion was located at or near the ring centroid for an electron-deficient aromatic system (F) (similar to the cation-π complex). By contrast, the anion tended to be located at the edge of the ring for an electron-rich π-system (Y) interacting with ring plane hydrogen atoms. A lone electron pair from the anion can interact with a non-bonding π-orbital in such a way as to provide intermediate geometry (known as a σ-type complex) [246].

An additional stabilising force involves π-systems (known as XH-π interaction); this has been described between methylene groups (-CH2-) (initially belonging to proline but extended to any X-H group) and peptide and protein π-orbitals, following observations of phenol-benzene and ammonia-benzene complexes in gas-phase [247]. Such force was defined as being a kind of hydrogen bond operating between a soft acid XH and a soft base π-system (not limited to aromatic moieties); it was studied from a supramolecular point of view, as was its impact on crystallographic systems [248].

A special case regarding XH-π interaction concerns a proline and an aromatic residue, this being critical for Y and W over F, due to additional electron density on the ring. This is due to proline’s unique role in protein folding, where an aromatic residue’s proximity to proline induces a conformational change from *cis*- to *trans*-proline. The particular non-covalent force’s nature has been described regarding local and tertiary structure, in protein-protein interactions as well as protein assemblies [249]. Kumar and Balaji’s [250] data-mining in two protein structure datasets determined CH-π features involving aa prevalence, propensities and location. Despite all these non-covalent forces being “weak”, they cannot be ignored since they are present in a protein or peptide in huge amounts so their final effect is comparable to that of covalent bonds [251].

Our group found that thrombospondin-related anonymous protein (TRAP)-derived cHABP 3287/89 (^241^TASCGVWDEWSPCSVTCGKGGTRSRK^265^) formed part of a β-sheet (A) structure, typical in thrombospondin-related (TSR) domains. It established multiple H-bonds with the backbone and side chain of aa located in the anti-parallel β-strand (B) structure. Stacked planar cationic guanidinium groups (R) and aromatic groups (W) were stabilized by multiple cationic-π interactions forming a continuous positively-charged surface containing a 20 Å-long groove-like structure, supposedly to receive negatively-charged ligands, such as heparin and heparan sulphates [252].

**Figure 5:**
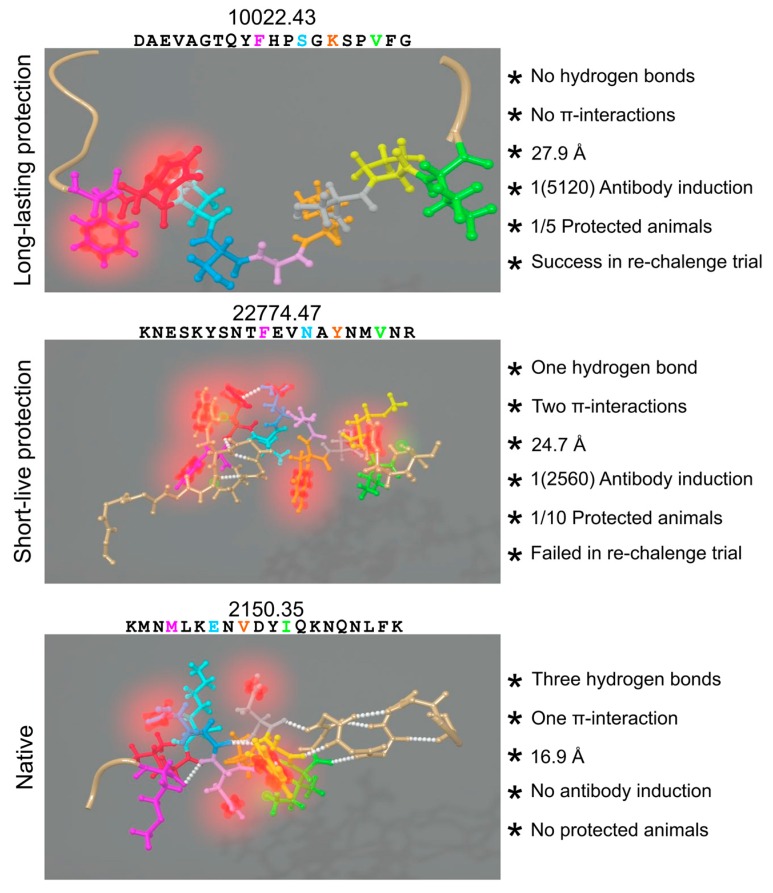
Intramolecular forces' influence: the peptide’sequence and 3D-structure is shown on the left, represented by balls and sticks. Residues, fitting into HLA-DRβ PBR coloured the same as in Figure 3: H-bonds in white spheres, π-systems in blurred red and interacting hydrogens in blurred green. Structural and immunogenic features of the nine residues from cHABPs and mHABPs interacting with the PBR are shown on the right. Figure based and adapted from [253]. Reproduced with permission from Patarroyo ME, *Biochem. Biophys. Res. Commun.*; published by Elsevier, 2017.

Our group recently published findings regarding the correlation between non-covalent forces’ presence (or absence) and the immunological behavior of several peptides used as vaccine candidates (Figure 5) [253]. The study concluded that intramolecular π-interactions and H-bonds in the PBR prevented PPIIL formation and their perfect fit into MHC class II molecules therefore induced different immune responses or none at all. These peptides became non-immunogenic and non-protection inducers when there was Aro residue upstream or downstream of any proline, completely distorting PPII_L_ conformation. Special care has thus to be taken into account when designing vaccine components involving π-CH interactions, as well as π-π, π-cationic, π-anionic, π-interactions and π-s interactions.

## 7. Peripheral Flanking Residues (PFR)

MHC recognition of peptides is not limited to the residues fitting into the PBR groove but also to the residues located before and after this important region, since this can influence MHC-peptide interactions, TCR recognition and antigen presentation and/or processing. Over the last two decades, several groups have proposed that these peripheral flanking residues (PFR) are crucial for inducing MHC-antigen-TCR complex formation and activity [254,255,256].

Nelson et al. established HEL 52–61 as the single minimal epitope capable of interacting with murine MHCII I-Ak molecule while studying the immune response driven by hen egg white lysozyme antigen (HEL) [257,258,259,260]. Furthermore, antigen-MHCII complex stability (measured by this complex’s stability during SDS/PAGE assays) became remarkably increased by adding residues to either end of the HEL 52–61 sequence, as did binding strength (affinity in competition assays). This led to proposing that, while HEL 52–61 contained the residues responsible for immunodominance, the PBR flanking residues were also needed at the ends of the antigen to make the additional contacts necessary for complex stabilization. When peptides containing HEL 52–61 were used for measuring peptide-MHCII complex lifespan it became clear that antigen-presenting cells (APCs) discriminated between antigen-MHCII complexes, as shown by differences arising as time elapsed. The study also suggested that peptide binding strength in the PBR was determined by the aa in p1 and backbone contacts made by N-terminal PFR [258].

Interestingly, Carson et al. [261] found a stronger T-cell response to antigens (including PFRs) when working with peptides (including the HEL 52–61 immunodominant epitope) and assessing direct mouse T-cell TCR recognition of PFR. These results highlighted PFRs’ immunological significance and implications (negative or positive) for immuno-regulation, peptide-based immunotherapy or vaccine development, since PFRs could hinder or unmask epitopes, thereby making them immunologically silent or more accessible to the immune response [261,262].

Based on the previous results, Godkin et al. investigated PFR preferences and whether such preferences were related to antigen processing events [263]. A pool of natural and synthetic peptides was thus constructed and exposed to several HLA-DR molecules to evaluate binding affinities, aa preference in different peripheral positions and TCR recognition. A preference for acidic residues at the antigen’s N-terminal as well as for basic residues at the antigen’s C-terminal was found and the results showed that such preferences were not related to antigen origin or MHC class II allele family.

Recent studies have shown PFRs’ intimate relationship with conformational stability, PFR length with increased binding affinity and how their N- and C-termini affect TCR binding ability and CD4+ T-cell activation and response as well as T-cell repertoire composition, thus suggesting novel applications regarding CD4+ T-cell peptide vaccine development and diagnostics [254,264,265].

Our recent findings involving ^1^H-NMR structural analysis and physico-chemical features regarding the effects of peripheral flanking residues on peptide-based malaria vaccine development [266] have demonstrated that −p2 (N-terminus) and p10 (C-Terminus) directly influence the immunological behavior of 20 mer-long peptides when used as vaccine candidates. MHCII’ F51α, A52α, S53α, F54α and V85β were the main contacts residues for the aa located in −p2, whilst MHCII’ A68α, N69α, I72α, D57β, W61β and L67β were the main aa contacting p10. These contacts supported well-studied N-terminal preference for acidic aa and C-terminal preference for basic aa regarding every antigen for proper immune response triggering. These differences enabled differentiating between three functionally-relevant groups: those inducing long-lasting protective memory, short-lived protective memory or non-protection-inducing but high antibody level producers.

**Figure 6:**
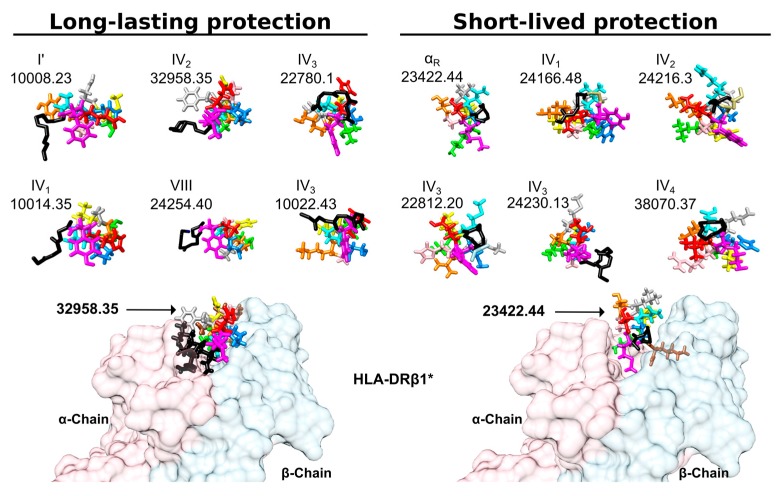
PFR beta structure: the front view shows long-lasting protective and short-live protection inducers. The colour code is the same as that used in Figure 3. Black sticks highlight the backbone of N-terminal peripheral region residues forming β-turn or α-helixes. Note that most structures were left-hand orientated suggesting an interaction with the HLA-DR α-chain to collaborate in stabilising this complex. Also note the total absence of the most frequent β-turn types, such as I, II and II’.

Our group’s studies ranging beyond N- or C-terminal flanking regions’ aa preference for any peptide vaccine candidate are currently geared to finding whether such regions have a preferred secondary structure. Several vaccine candidates’ two flanking regions have elicited the same 3D-structure (Figure 6, submitted data): the N-terminal peripheral region pointing downwards towards the MHCII and the C-terminal peripheral region pointing upwards towards the TCR. On-going analysis concerns both peripheral flanking regions’ secondary structure preference; a specific β-turn in the N-terminal section has been established so far (submitted data).

The idea behind the aforementioned search was that MHCII-peptide-TCR complex formation leads to the PBR’s nine aa making contact with both proteins (MHC and TCR), and residues located before and after this region, implying their semi-conserved structure due to well-known MHCII-TCR architecture and these two regions’ high importance, as recognized by several research groups during the last two decades.

## 8. Concluding Remarks

**Figure 7:**
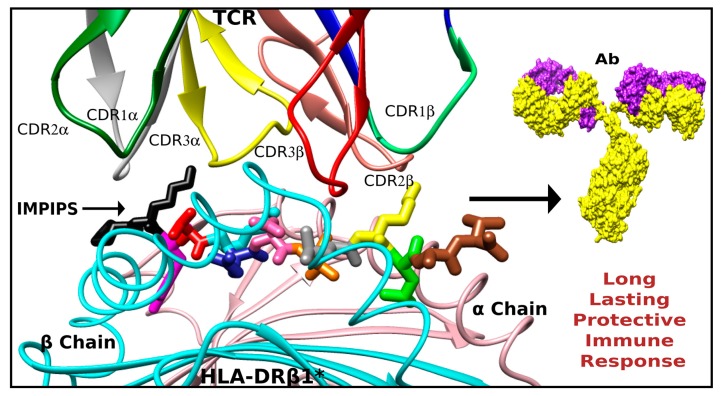
The physical and chemical features which must be taken into account for a peptide-based vaccine candidate design. A perfect fit of the tertiary complex formed by the MHC class II (bottom, ribbon representation), the IMPIPS (middle, stick representation colored as in the Figure 3) and the TCR (displaying the complementary determinant regions loops on each chain in ribbon representation) is the only way to induce a long-lasting and protective immune response.

Synthetic peptides can be used for many functions since they have a single tremendous advantage over other biological resources in that they are chemically defined. Different sequences having different properties can be combined, their structures can be easily modulated or refined by aa replacement and biological function can be mimicked, inhibited or increased [267]. Such aspects can be exploited in a subunit-synthetic peptide-based vaccine approach. We have found that synthetic peptides having conserved *P. falciparum* protein sequences fulfilling different biological functions have been non-immunogenic or protection-inducing against experimental challenge in the *Aotus* monkey model. However, careful aa changes in cHABP sequences have improved peptide affinity for MHCII molecules and converted them into immunogenic protection-inducers in the *Aotus* monkey model. Replacing aa must consider each native conserved (cHABP) sequence’s stereo and physiochemical characteristics regarding their modified analogue (mHABP) sequences and MHCII PBR sequences (Figure 7).

The following precepts must be observed in subunit vaccine development (i.e., IMPIPS design): 26.5 ± 1.5 Å is the ideal distance between the first (p1) and ninth (p9) aa of the peptide region fitting into the PBR (nb, intra H-bonds and intra π-interactions must be avoided); aa volume and charge when replacing p4, p6 and p9 residues must be selected according to the pockets’ sequence in each PBR; modified peptides (mHABP) must have a polyproline II-like (PPIIL) structure and their backbone O and N atoms orientated to establish H-bonds with PBR residues; p3 and p7 residues must have p3χ^1^, and p7χ^1^ angle *gauche*^+^ orientation, allowing appropriate rotamer orientation to interact with TCR residues; and N-terminus PFR outside the PBR must have specific β-turn structures.

We would suggest that using synthetic peptides and following the aforementioned rules enables the feasibility of vaccines based on synthetic peptides as subunits to be introduced against infectious diseases, as well as microbe-associated or induced-cancers, in a relatively short time (Figure 7).

## Figures and Tables

**Table 1:** Synthetic peptides as vaccines.

Name	Disease	Agent	Model & Outcome	Ref.
	Diphtheria	*Corynebacterium diphtheriae*	Guinea pigs became protected	[45]
	Foot-and-mouth disease	*Foot-and-mouth disease virus*	Neutralising antibodies induced in rabbits and guinea pigs	[46]
	Foot-and-mouth disease	*Foot-and-mouth disease virus*	Cattle became protected	[47]
	Malaria	*Plasmodium berghei*	Mice became protected	[38]
	Gastroenteritis	*Simian rotavirus*	Neutralising antibodies induced in rabbits	[48]
	Acquired immune deficiency syndrome	*Human immunodeficiency virus*	Neutralising antibodies induced in rabbits	[49]
First multi-epitope synthetic antimalarial vaccine	Malaria	*Plasmodium falciparum*	*Aotus* monkeys became protected	[39]
SPf66	Malaria	*Plasmodium falciparum*	Humans became protected	[11]
SPf66	Malaria	*Plasmodium falciparum*	Safe and highly-immunogenic in humans	[50,51,52]
SPf66	Malaria	*Plasmodium falciparum*	Protective efficacy (30–40%) in semi-immune populations	[12,53,54,55]
	Haemorrhagic enteritis, Myocarditis	*Canine parvovirus*	Dogs became protected	[56]
	Malaria	*Plasmodium vivax*	Parasite development in the mosquito was blocked	[57]
	Anthrax	*Bacillus anthracis*	Rabbit became protected	[58]
Fusion peptide	Influenza	*Influenza A virus*	Mice became protected	[59].
	Swine fever	*Classical swine fever virus*	Neutralising antibodies induced in pigs	[60]
CIGB-228	Cervical intraepithelial neoplasia	*Human papilloma virus*	Lesion regression and HPV clearance	[61].
IMPIPS	Malaria	*Plasmodium falciparum*	*Aotus* monkeys became protected	[62,63]
